# Effects of Sandblasting at Different Angles Combined with Subsequent Acid Pickling on the Microstructure and Surface Properties of SLM-Formed Ti-6Al-4V Alloy

**DOI:** 10.3390/mi17080890

**Published:** 2026-07-25

**Authors:** Yuanyuan Xie, Lei Li

**Affiliations:** School of Mechanics and Aeronautics, Inner Mongolia University of Technology, Aimin Sub-District, Xincheng District, Hohhot 010051, China; xieyy26411@163.com

**Keywords:** SLM Ti-6Al-4V, surface modification, sandblasting, acid etching, surface roughness

## Abstract

Ti-6Al-4V alloy possesses excellent specific strength, corrosion resistance, and biocompatibility, rendering it widely applicable in aerospace, marine engineering and biomedical fields. Selective laser melting (SLM) serves as an effective technique for manufacturing complex Ti-6Al-4V components. However, SLM-formed specimens generally suffer from surface defects such as high surface roughness, adhered powders, spheroidized particles, and localized spatter, which degrade their service performance and limit further practical applications. Therefore, effective surface modification is urgently required. This work systematically explores the synergistic effects of sandblasting at various angles followed by acid pickling on the surface characteristics of SLM-formed Ti-6Al-4V alloy. The SLM Ti-6Al-4V samples were first treated by sandblasting at different impact angles and then subjected to acid pickling. Material mass loss, micro-morphology, surface roughness, contact angle, surface microhardness, abrasive-particle embedment and surface residual stress were measured and analyzed. The results show that sandblasting angle exerts a remarkable influence on material removal behavior, abrasive-particle embedment and near-surface mechanical response. Scanning electron microscopy (SEM) observations indicate that sandblasting at different angles can not only effectively eliminate surface-adhered powders, but also generate impact pits, cutting grooves, and ploughing marks whose morphologies vary with sandblasting angles. The subsequent acid pickling process further removes loose particles and sharp protrusions, and promotes the formation of microscale surface structures. Benefiting from the combined effects of mechanical sandblasting and chemical acid pickling, the alloy samples exhibit substantially reduced surface roughness and enhanced surface wettability. Meanwhile, sandblasting induces work hardening and thus increases surface microhardness and surface residual stress, while acid pickling regulates surface morphology and the state of the work-hardened layer to a certain degree. Overall, this study provides an economical, efficient, and industrially feasible composite surface modification approach to reduce surface roughness, enhance hydrophilicity, and tailor surface hardness of SLM Ti-6Al-4V alloy.

## 1. Introduction

Ti-6Al-4V alloy possesses excellent specific strength, corrosion resistance, and biocompatibility, and thus has been widely adopted in aerospace, marine engineering, and biomedical fields [1,2,3,4,5]. However, conventional processing techniques are overly complicated for components with intricate geometries, resulting in low material utilization and high production cost [6,7]. Selective laser melting presents distinct advantages in preparing complex and customized alloy structures and plays an irreplaceable role in complex engineering manufacturing. This technique enables the fabrication of complex components and rapid repair of damaged parts, which substantially cuts costs and time and facilitates the advancement of aerospace and medical fields [8,9]. SLM-formed Ti-6Al-4V exhibits quasi-equivalent static mechanical properties to wrought parts, making it one of the most promising materials for SLM applications [10]. Nevertheless, as-built SLM components suffer from inherent defects, including high surface roughness, high residual stress, and severe powder adhesion caused by partially melted spheroidized particles, which severely deteriorate their service performance [11,12,13]. In addition, surface wettability and microhardness are also key indicators governing the comprehensive performance of Ti-6Al-4V in practical service [14]. Therefore, synergistic regulation of the surface morphology and physical properties of SLM Ti-6Al-4V alloy through various processes has become a research focus in the field of surface modification [15].

Among diverse surface treatment techniques, sandblasting is a classical physical modification method and is widely applied to mechanical material removal and surface contouring of metallic materials. In recent years, numerous studies have been carried out on sandblasting for titanium alloys and other metallic materials, verifying its capability to improve specific properties. Bagehorn et al. compared several mechanical treatment methods and clearly demonstrated that sandblasting can effectively eliminate macroscopic stair-step defects and spheroidized particles formed during SLM. The removal of these stress concentration sites, which serve as initiation sources for fatigue cracks, enhances the high-cycle fatigue life of SLM Ti-6Al-4V by orders of magnitude [16]. Cerri et al. reported that, during sandblasting, the Vickers microhardness of the surface layer significantly increased because the surface material underwent work hardening under microbead impact. The formation of a hardened surface layer further improves surface wear resistance and resistance to local deformation [17]. Wennerberg et al. performed a comparative investigation into sandblasting with abrasives of different particle sizes and found that microscale rough morphologies constructed under specific parameters can effectively increase surface free energy, and thereby significantly enhance the intrinsic wettability and liquid spreading capacity of titanium alloy surfaces [18]. Kim et al. employed high-energy impact with hard particles such as Al_2_O_3_ or SiO_2_ to construct porous rough surfaces with large specific surface areas on titanium substrates. The resulting mechanical interlocking structure effectively strengthens the adhesion between subsequent coatings or osseous tissue and the substrate [19]. Fang and Chuang investigated stainless steels, weathering steel, brass, and 6063 aluminum alloy and found that different materials exhibited distinct erosion rates and surface damage morphologies under abrasive impact [20]. For low-carbon steel, Chander et al. reported that the material-removal mechanism changed among microcutting, indentation, and mixed modes depending on the impact angle, while increasing the blasting pressure and angle enhanced the compressive residual stress and subsurface hardness [21]. In SLM-fabricated Co–Cr–W alloy, sandblasting modified the surface roughness, induced surface hardening, and improved liquid-droplet adhesion, confirming that additively manufactured alloys also exhibit a pronounced mechanical and wetting response to abrasive treatment [22].

However, single sandblasting treatment still has limitations. Shinonaga et al. found that abrasive particles tend to embed in the surface of SLM Ti-6Al-4V after sandblasting. Such abrasive embedment may change the local chemical composition and physical integrity of the surface [23]. Baleani et al. directly demonstrated that high-energy impact not only generated microcracks on titanium alloy surfaces but also left deeply embedded alumina fragments that acted as stress concentration zones. Under cyclic loading or friction service conditions, these zones are prone to crack initiation and eventually cause premature component failure [24]. Pellegrini et al. also pointed out that high-energy impact inevitably led to partial deep embedment or retention of hard abrasives on the titanium alloy substrate surface, which may not only induce local stress concentration but also change the local electrochemical potential and consequently trigger micro-galvanic corrosion [25]. Peñuela-Cruz et al. demonstrated that blasting pressure was the dominant parameter governing the roughness and hardness of magnesium sheets, although excessive impact intensity could also generate surface cracks and voids [26]. Given the drawbacks of standalone physical deformation processes, it remains an engineering challenge to retain the favorable macroscopic rough framework generated by sandblasting while effectively eliminating residual highly stressed hardened layers. As a highly selective post-treatment method, acid pickling can effectively address the above issues.

Different from mechanical abrasive impact, acid pickling constructs micro-morphology primarily through chemical etching on the substrate. When applied as a key post-treatment following sandblasting, it plays an irreplaceable role in surface microstructural reshaping. Akbas et al. showed that the combination of optimized sandblasting pressure and acid etching could effectively homogenize the inferior surface morphology of additively manufactured titanium alloys, rendering their bioactivity comparable to that of conventional clinical bone implants [27]. Orsini et al. demonstrated that specific mixed-acid systems, such as HF/HNO_3_, can effectively remove residual heterogeneous hard particles after sandblasting and facilitate the in situ formation of a uniform and dense TiO_2_ passive film, thereby significantly improving corrosion resistance [28]. Morphological evolution analysis by Adhitya et al. further indicated that acid pickling exerted a prominent effect on cutting sharp micro-protrusions and smoothing surface irregularities induced by sandblasting and can passivate geometric stress concentration sites to a certain extent [29]. Moreover, combined mechanical and chemical treatments have been investigated for different substrates. Geng et al. found that subsequent acid pickling and passivation partially restored the corrosion resistance of sandblasted 316L stainless steel while largely retaining the increased surface hardness [30].

Although extensive research has confirmed that combined sandblasting and acid pickling can effectively improve the surface quality of SLM-fabricated titanium alloys, most existing studies have focused on optimizing process parameters such as blasting pressure, abrasive particle size and type, acid solution composition, and pickling duration. By contrast, the sandblasting impact angle has received far less systematic investigation. This parameter fundamentally determines the relative contributions of the normal and tangential components of abrasive velocity: at steep impact angles, the normal component dominates, and the surface primarily undergoes plastic indentation and impact pit formation accompanied by pronounced near-surface plastic deformation; at shallow impact angles, the tangential component prevails, shifting the dominant material removal mechanism toward micro-cutting and ploughing. This suggests that varying sandblasting angles not only yield distinct material removal rates and work hardening levels, but also produce fundamentally different initial surface geometries and near-surface mechanical states. These differences are likely to further modulate the selective dissolution behavior during subsequent acid pickling, ultimately manifesting as variations in surface roughness, wettability, microhardness, and residual stress.

However, a systematic evaluation linking sandblasting angle to the subsequent acid pickling response is still lacking, and the mechanism through which impact angle mediates the coupling between mechanical impact and chemical etching remains poorly understood. Clarifying how impact angle shapes the initial surface state, and how this initial state in turn governs surface evolution during the ensuing pickling stage, is therefore critical to establishing a property-oriented basis for selecting appropriate surface modification processes.

In this study, as-built SLM Ti-6Al-4V specimens were sandblasted at 30°, 60°, and 90° with all other processing parameters held constant, followed by an identical acid pickling treatment. The resulting variations in mass loss, surface morphology, roughness, wettability, microhardness, and residual stress were systematically characterized. By establishing the correlation among impact angle, initial surface state, and post-pickling performance, this study reveals the angle-dependent evolution of surface properties and provides a property-oriented basis for selecting suitable sandblasting conditions for additively manufactured Ti-6Al-4V components.

## 2. Materials and Methods

### 2.1. Specimen Preparation

**Figure 1:**
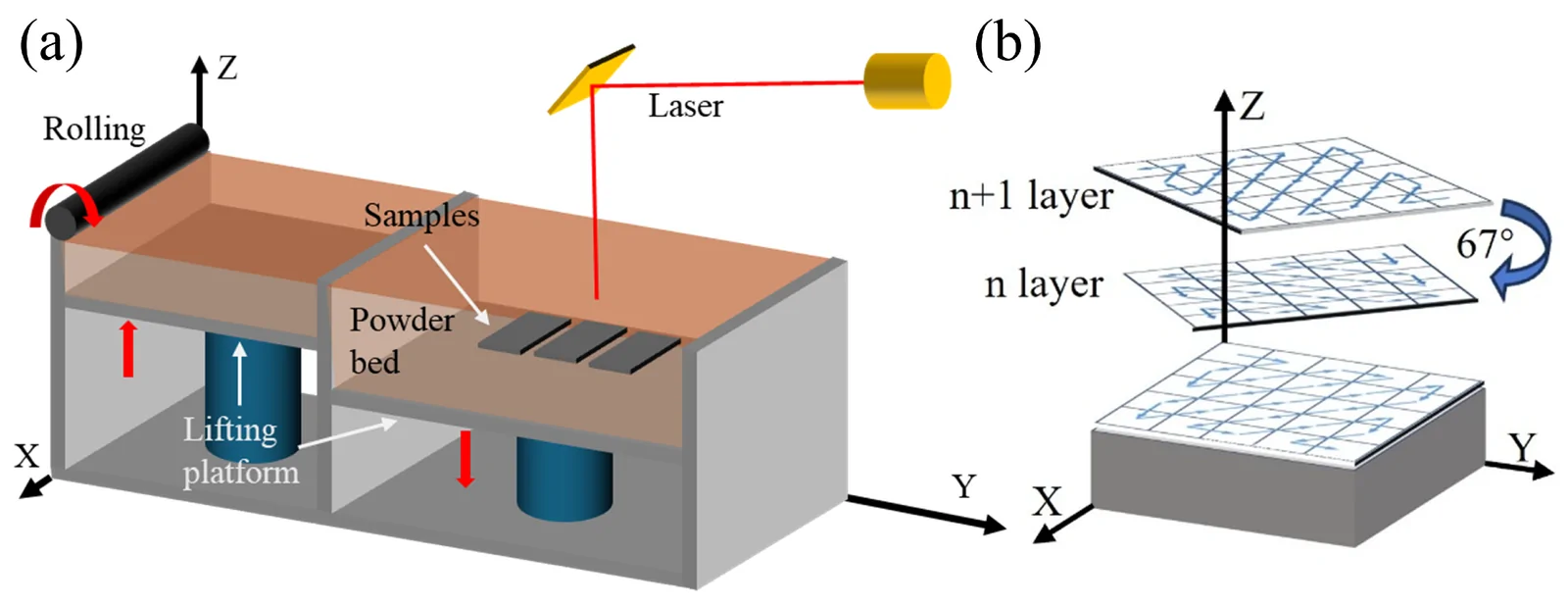
Schematic diagram of specimen forming: (**a**) SLM process; (**b**) scanning strategy.

The schematic diagram of specimen forming is shown in Figure 1. The specimens were fabricated using an EOS-M290 selective laser melting system manufactured by EOS GmbH, Maisach, Germany. Ti-6Al-4V titanium alloy powder was adopted as the raw material, and its chemical composition is listed in Table 1.

The powder particle size was distributed in the range of 15–53 μm. To avoid oxidation and burning loss of Ti-6Al-4V alloy during high-temperature fabrication, the forming chamber was evacuated before printing and then filled with high-purity argon to create an inert protective atmosphere. During forming, the oxygen concentration inside the chamber was maintained below 0.1%. The layer thickness was set to 0.06 mm. For solid bulk regions, the processing parameters were set as follows: laser power of 340 W, scanning speed of 1250 mm/s, laser spot diameter of 0.100 mm, and hatch spacing of 0.120 mm.

**Figure 2:**
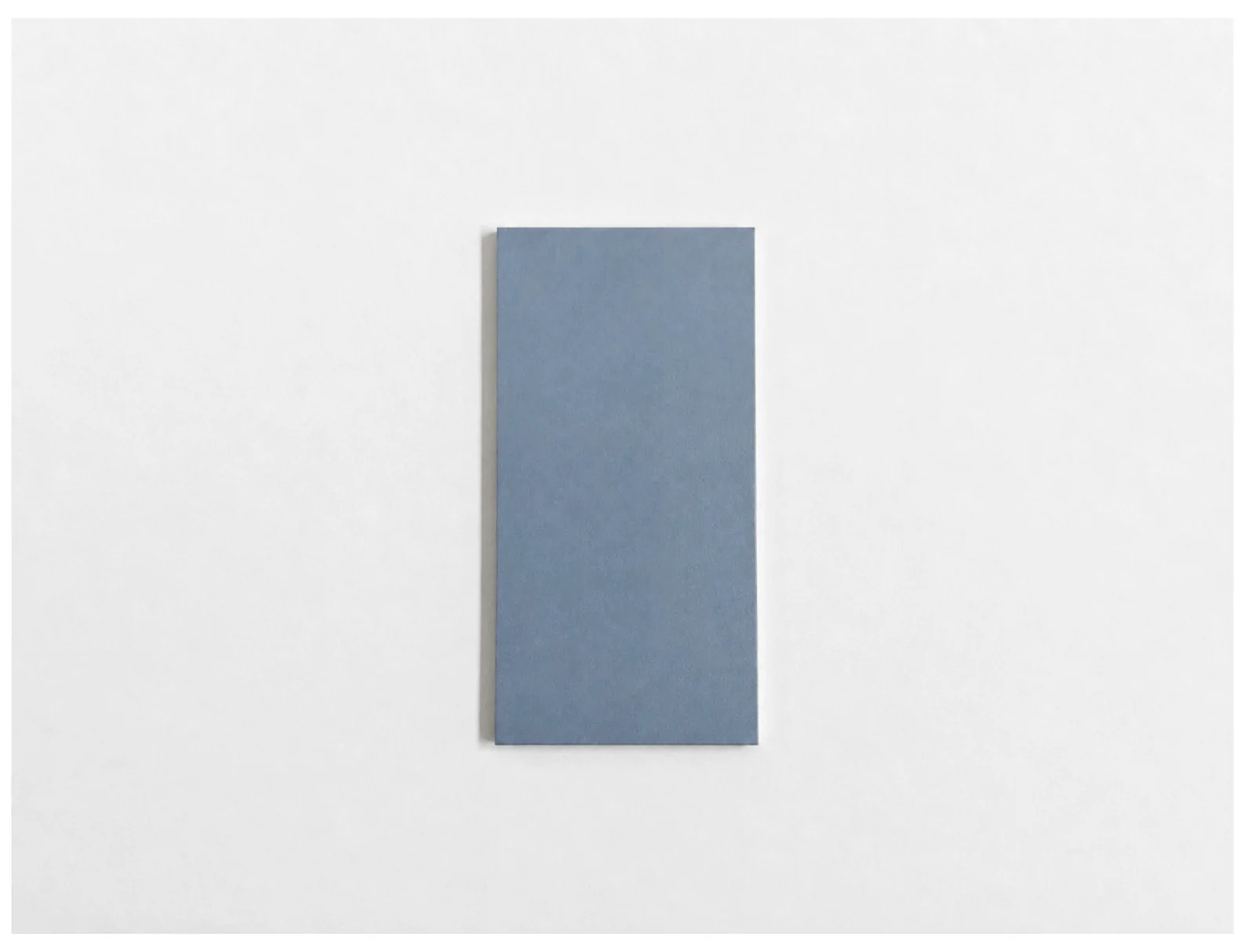
SLM-formed Ti-6Al-4V specimen.

A stripe scanning strategy was adopted for the interior regions. The stripe width was 5.000 mm with a zigzag scan path. The initial scanning angle was 30°, and the rotation angle between successive layers was 67°. Two contour scans were performed, with a contour spacing of 0.040 mm and a beam offset of 0.045 mm. The non-solid support regions were formed using a laser power of 100 W, a scanning speed of 600 mm/s, and a laser spot diameter of 0.100 mm. Upon completion of fabrication, samples were detached from the base plate, and residual supports were removed prior to the combined sandblasting and acid pickling treatment. A total of 21 identical specimens were prepared for subsequent treatments and experiments. The actual specimen is shown in Figure 2.

### 2.2. Specimen Treatment

#### 2.2.1. Sandblasting Treatment

**Figure 3:**
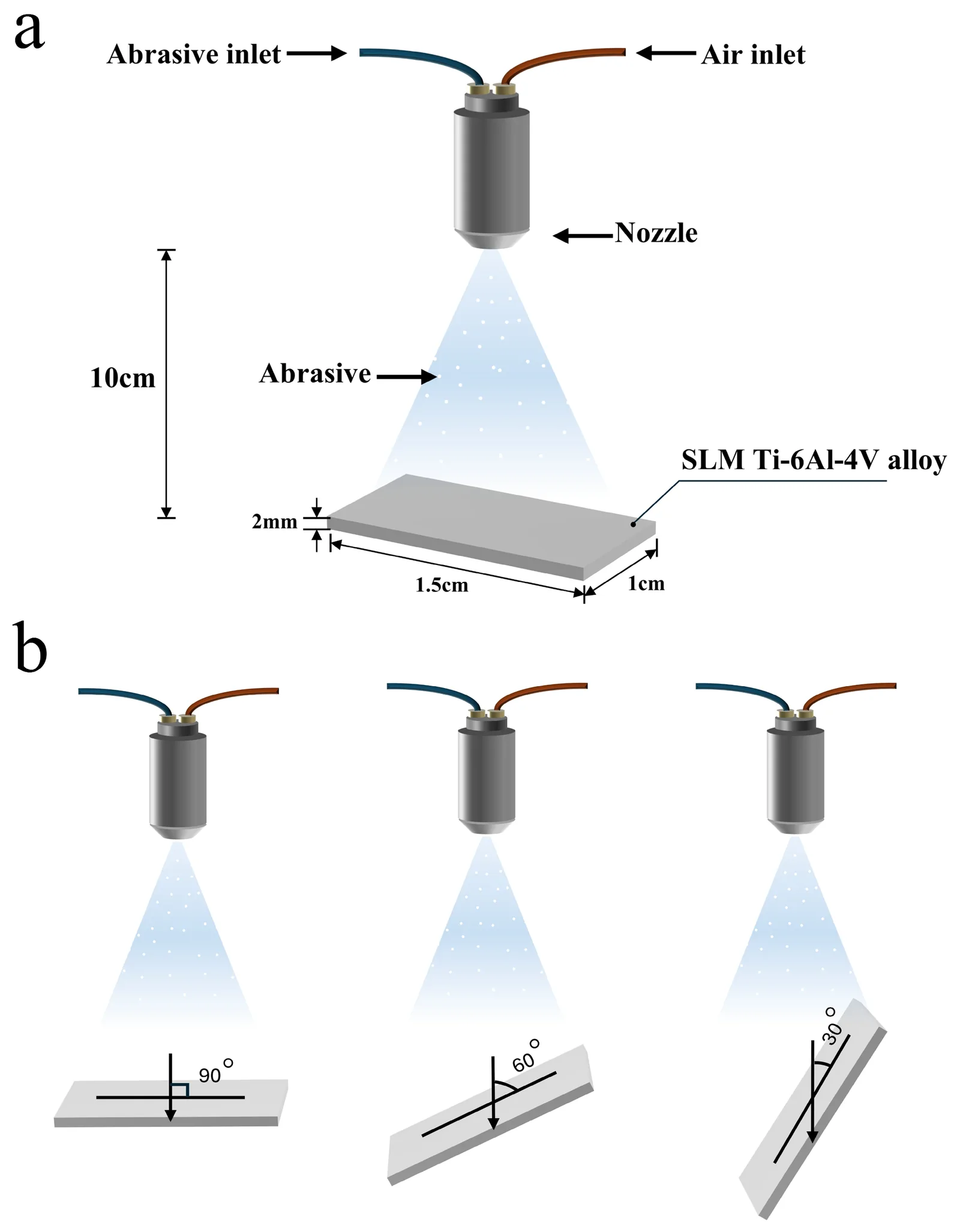
(**a**) Schematic diagram of sandblasting; (**b**) angle adjustment method.

The schematic diagram of sandblasting treatment is shown in Figure 3. All SLM Ti-6Al-4V samples were subjected to surface impact treatment at room temperature using a dry sandblasting machine (AX-P3, AIXIN, Tianjin, China). Quartz sand (SiO_2_) particles with a Mohs hardness of 7 and a particle size of 40–70 mesh were adopted as the abrasive medium. Quartz sand was selected based on considerations of both surface contamination risk and engineering applicability. Al_2_O_3_ particles can become embedded in titanium alloy surfaces during blasting and are difficult to remove completely via conventional acid pickling owing to their high chemical stability, potentially leading to persistent Al- and O-bearing contamination. In addition, quartz sand is low-cost, readily available, and exhibits higher cutting efficiency and material removal capacity than spherical glass beads, making it more economically viable for large-scale surface treatment of additively manufactured Ti-6Al-4V components. Before sandblasting, the abrasive exhibited irregular shapes with sharp edges, which facilitated cutting, ploughing, and local impact during blasting. The blasting pressure was set to 0.4 MPa, corresponding to an abrasive feed rate of 460 g/min. The blasting distance was 10 cm, with full surface coverage and a single blasting duration of 10 min. To investigate the effect of sandblasting angle on surface modification, three angles were set as 30°, 60°, and 90°. The sandblasting angles of 30°, 60°, and 90° were chosen with reference to prior studies on angle-dependent abrasive erosion and surface modification of ductile metallic materials. These three angles correspond to three distinct particle impact regimes. At 30°, the tangential component of abrasive velocity is relatively dominant, promoting cutting, ploughing, and material removal. At 90°, the normal component reaches its maximum, primarily inducing plastic indentation, impact pit formation, and near-surface deformation. The 60° condition represents an intermediate regime where tangential material removal and normal plastic deformation act simultaneously. Accordingly, the selected angle set was designed to capture the transition of the surface modification mechanism from tangential-shear-dominated impact to normal-impact-dominated deformation.

After sandblasting, the specimens were immediately removed, ultrasonically cleaned, and fully dried to remove loosely attached abrasive particles and surface contaminants, thereby providing a stable initial surface state for the subsequent acid pickling and characterization. To ensure the reproducibility of the sandblasting process, all specimens were treated using the same dry sandblasting apparatus and the same batch of SiO_2_ abrasive with a consistent particle size range. Three independent specimens were prepared for each treatment condition, and an identical operating protocol was followed throughout the experiments to minimize variations in abrasive delivery and impact exposure. In addition, all specimens were fabricated using the same SLM equipment, powder batch, and processing parameters to reduce disparities in their initial surface states. Process reproducibility was controlled through strict regulation of macroscopic blasting parameters and repeated treatment under identical conditions.

#### 2.2.2. Acid Pickling Treatment

**Figure 4:**
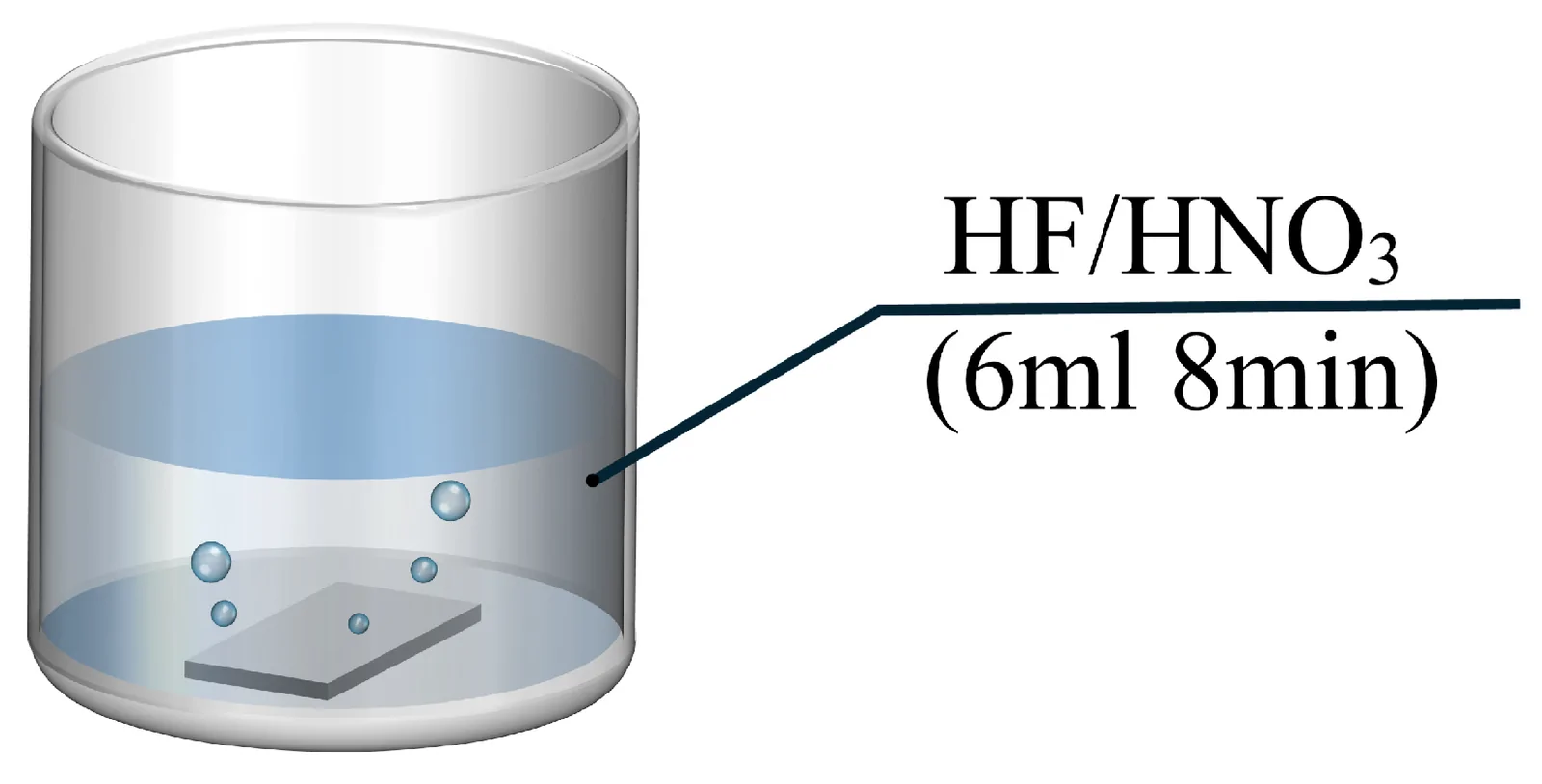
Schematic diagram of acid pickling.

Finally, acid pickling was performed at room temperature using a mixed acid solution consisting of HF, HNO_3_ (Kemao, Tianjin, China) and deionized water at a volume ratio of 1:4:15. As shown in Figure 4, a volume of 6 mL of acid solution was applied to each group to ensure consistent etching conditions, and the pickling duration was set to 8 min. The acid solution composition and treatment duration were determined based on well-established HF-HNO_3_ pickling procedures for Ti-6Al-4V, combined with acid-pickling experience accumulated in our previous work. These parameters were kept identical across all sandblasted groups. After the reaction, samples were promptly taken out and repeatedly cleaned with deionized water via ultrasonication. Finally, all treated specimens were dried in a drying oven. All specimens were labeled according to blasting angle and post-pickling treatment: original, SB30, SB60, SB90, SLA30, SLA60, and SLA90. The specific grouping is shown in Table 2.

### 2.3. Test Methods

#### 2.3.1. Mass Loss Test

Mass loss tests were conducted to quantify the material removal behavior of SLM Ti-6Al-4V under different blasting angles and acid pickling treatments. An electronic balance (BCE224I-1S, Sartorius AG, Goettingen, Germany) with a mass resolution of 0.1 mg was used for weighing. Before testing, the specimens were ultrasonically cleaned and fully dried to eliminate the influence of residual abrasives, acid solution, and reaction by-products. The mass before treatment, *m*_0_, and the mass after treatment, *m*_1_, were recorded, and the mass loss was calculated according to Equation (1):

*Δm* = *m*_0_ − *m*_1_ (1)

where *Δm* denotes the specimen mass loss. To minimize random errors, each group was weighed multiple times, and the average value was adopted as the final result. All samples were fully dried before weighing to avoid errors caused by residual moisture.

#### 2.3.2. Microhardness Test

The surface microhardness of the samples was measured using a manual Vickers microhardness tester (HV-1000, CVOKLY, Dongguan, China). A regular square-pyramidal Vickers diamond indenter was used, under an applied load of 100 gf, namely HV0.1, with a dwell time of 10 s. The diagonal lengths of each indentation were measured using the built-in reading microscope, and the corresponding hardness values were calculated accordingly. To ensure the representativeness of the test results and reduce the influence of local microstructural heterogeneity, indentations were made at 10 random positions on each sample surface. The spacing between adjacent indentations was kept at no less than three times the indentation diagonal length, so as to prevent mutual interference between plastic deformation and strain-hardened regions, thereby ensuring the independence of each test point. The Vickers microhardness was then calculated according to Equation (2):

(2) $$HV=\frac{2Fsin\left(\frac{136{}^{\circ}}{2}\right)}{d^{2}}$$

After unloading, the two diagonal lengths of each indentation, *d*_1_ and *d*_2_, were measured using the built-in optical microscope, and the mean diagonal length, *d*, was calculated as Equation (3):

(3) $$d=\frac{d_{1}+d_{2}}{2}$$

The final microhardness result was defined as the arithmetic mean of the 10 measurements.

#### 2.3.3. Three-Dimensional Morphology and Roughness Test

A laser confocal microscope (OLS4100, Olympus Corporation, Tokyo, Japan) was employed to observe the three-dimensional surface morphology and measure surface roughness of the samples. Measurements were carried out with a 20× objective lens, and the single measurement area was 645 μm × 656 μm. To obtain stable and reliable roughness parameters, Gaussian filtering was applied to the acquired surface morphology data, with the roughness cutoff wavelength λc set to 250 μm. Subsequently, the instrument analysis software (Olympus LEXT OLS4100, v3.1.10) was used to reconstruct the three-dimensional surface profiles from layered scanned images and calculate the corresponding surface roughness parameters.

#### 2.3.4. Micro-Morphology Observation and Embedded-Particle Quantification

The surface microtopography and chemical composition of the samples were analyzed using a field-emission scanning electron microscope (Apreo S LoVac, Thermo Fisher Scientific, Waltham, MA, USA). During testing, the surface morphology and local features of samples under different treatment states were mainly observed, and the composition of surface regions was analyzed. For SEM observation, the accelerating voltage was set to 15 kV, the working distance was 4.86 mm, and the beam current was 1.6 nA to acquire high-resolution and stable images. By comparing the surface micro-morphologies of samples from different treatment groups, the effects of sandblasting angle and subsequent acid pickling on grooves, impact pits, plastic deformation zones, residual particles, and acid-etched morphology on the SLM Ti-6Al-4V surface were analyzed.

The surface elemental composition was analyzed using energy-dispersive X-ray spectroscopy (EDS) mapping integrated with the SEM. Finally, post-processing software (v23.x, Thermo Scientific Pathfinder software, Waltham, MA, USA) was used to analyze the acquired X-ray count pulses, generating EDS spectra and quantitative tables to characterize the surface micro-morphological features and the changes in surface elements caused by different treatment processes.

On this basis, the SiO_2_ abrasive particles embedded in the surface after sandblasting were quantitatively analyzed. For each sandblasting condition, three independent specimens were selected, and ten non-overlapping SEM fields of view were randomly acquired from each specimen. All images were obtained at the same magnification and under identical imaging parameters. The visible embedded SiO_2_ particles on the surface were identified by combining SEM morphological features with the co-localized signals of Si and O elements, and their projected areas were measured using image software (ImageJ 1.54u7, Bethesda, MA, USA). A unified minimum equivalent diameter was set to exclude small regions below the reliable detection limit and image noise. For particles that were in contact with each other but could be reliably separated, their projected areas were measured separately; for connected regions that could not be reliably separated, the entire connected region was treated as a particle cluster.

The median projected area of independent particles with clear boundaries in each treatment group was used as the reference particle area, and the projected areas of both independent particles and particle clusters were converted into equivalent particle numbers. The equivalent number of embedded particles over the entire treated surface was estimated from the ratio of total projected particle area per field of view to the observed area, combined with the total treated surface area of the specimen. First, the average result of ten fields of view was calculated for each specimen; then, the mean value and standard deviation for each treatment condition were calculated from three independent specimens. The results are expressed as mean ± standard deviation, with the number of independent replicates being 3.

#### 2.3.5. Contact Angle Test

The surface hydrophilicity of the samples was evaluated using a contact angle measuring instrument (DSA25, KRÜSS GmbH, Hamburg, Germany) at a constant temperature of 20 °C. Deionized water was selected as the test liquid, and static contact angles were measured to characterize surface hydrophilicity. After the droplet was deposited on the sample surface, the instrument software was used for image acquisition and contact angle calculation. The ellipse fitting algorithm (Tangent-1) was selected as the contact angle fitting method to accurately identify the droplet contour and reflect local spreading behavior on rough surface microstructures. Five different positions were randomly selected on each sample for testing. For each measurement, 5 μL of deionized water was dropped, the droplet was allowed to stabilize for 5 s, and then images were acquired. The average value was taken as the final result.

#### 2.3.6. Residual Stress Test

Surface residual stresses were measured using an X-ray residual stress analyzer (PROTO LXRD, Proto Manufacturing, LaSalle, ON, Canada) based on the sin^2^ψ method. Measurements were performed along the X and Y directions of the specimen surface. The {213} diffraction plane of the α-Ti phase was selected using Cu Kα radiation (λ = 1.541838 Å) with a Ni filter. The tube voltage and current were set to 30 kV and 25 mA, respectively, and the nominal diffraction angle was 2θ = 142.00°. The ψ angle ranged from approximately −44° to 44°. The diffraction peaks were fitted using a Gaussian function, and the residual stress was calculated from the linear relationship between the lattice spacing and sin^2^ψ. Tensile stress was defined as positive, whereas compressive stress was defined as negative.

## 3. Results

### 3.1. Analysis of Mass Loss Results

**Figure 5:**
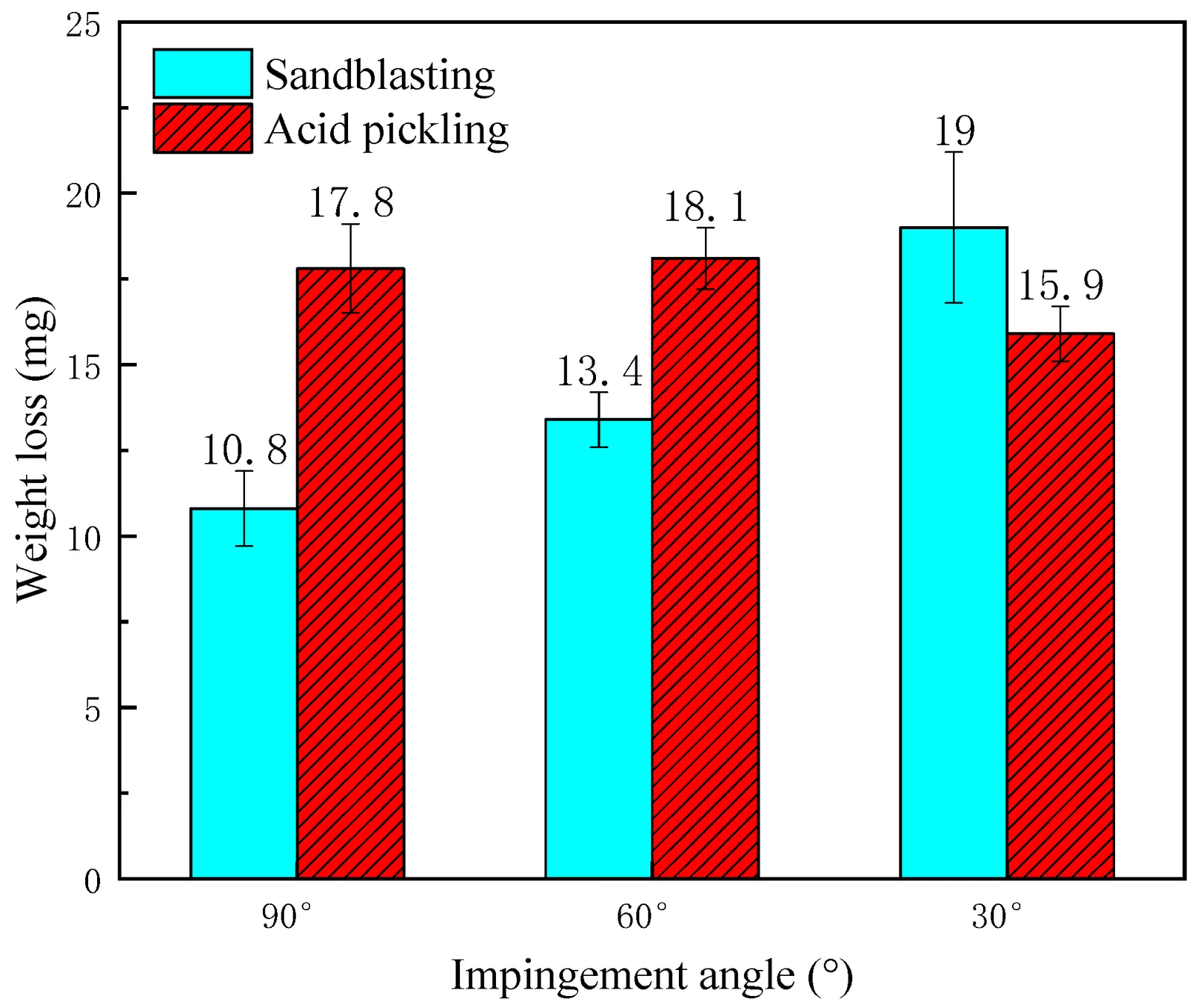
Comparison of mass loss at different sandblasting angles and comparison of mass loss after acid pickling at the corresponding sandblasting angles.

To reveal the effect of sandblasting angle on the surface modification behavior of SLM Ti-6Al-4V, mass loss tests were first performed on samples treated at different angles, as shown in Figure 5. With identical abrasive type, sandblasting pressure, sandblasting duration, and sandblasting distance, the samples treated at 30° exhibited the maximum mass loss, while those treated at 90° showed the minimum mass loss; the 60° group fell in between. This indicates that the sandblasting angle significantly affects the interaction between abrasive particles and the titanium alloy surface [31]. At a small sandblasting angle, abrasives carried a large tangential force along the sample surface, which primarily induced ploughing, scraping, and cutting actions. Therefore, adhered powders, sharp peaks, and partial substrate material were more easily removed, leading to greater mass loss. When the sandblasting angle increased to 90°, the abrasive particles mainly acted on the surface through normal impact, which weakened material removal. The surface response was dominated by indentation and plastic deformation accompanied by work hardening, thus yielding the lowest mass loss [32].

After further acid pickling, the mass loss differences among groups with different angles became insignificant, indicating that the overall material removal during acid pickling was mainly controlled by acid concentration and pickling duration in this work [33]. The sandblasting angle exerted only a minor influence on the overall dissolution amount during the subsequent acid pickling stage. These results showed that different sandblasting angles altered the surface material removal mode and near-surface deformation state in the first stage, thereby laying the foundation for subsequent changes in roughness, wettability, and microhardness.

### 3.2. Analysis of Microhardness Results

**Figure 6:**
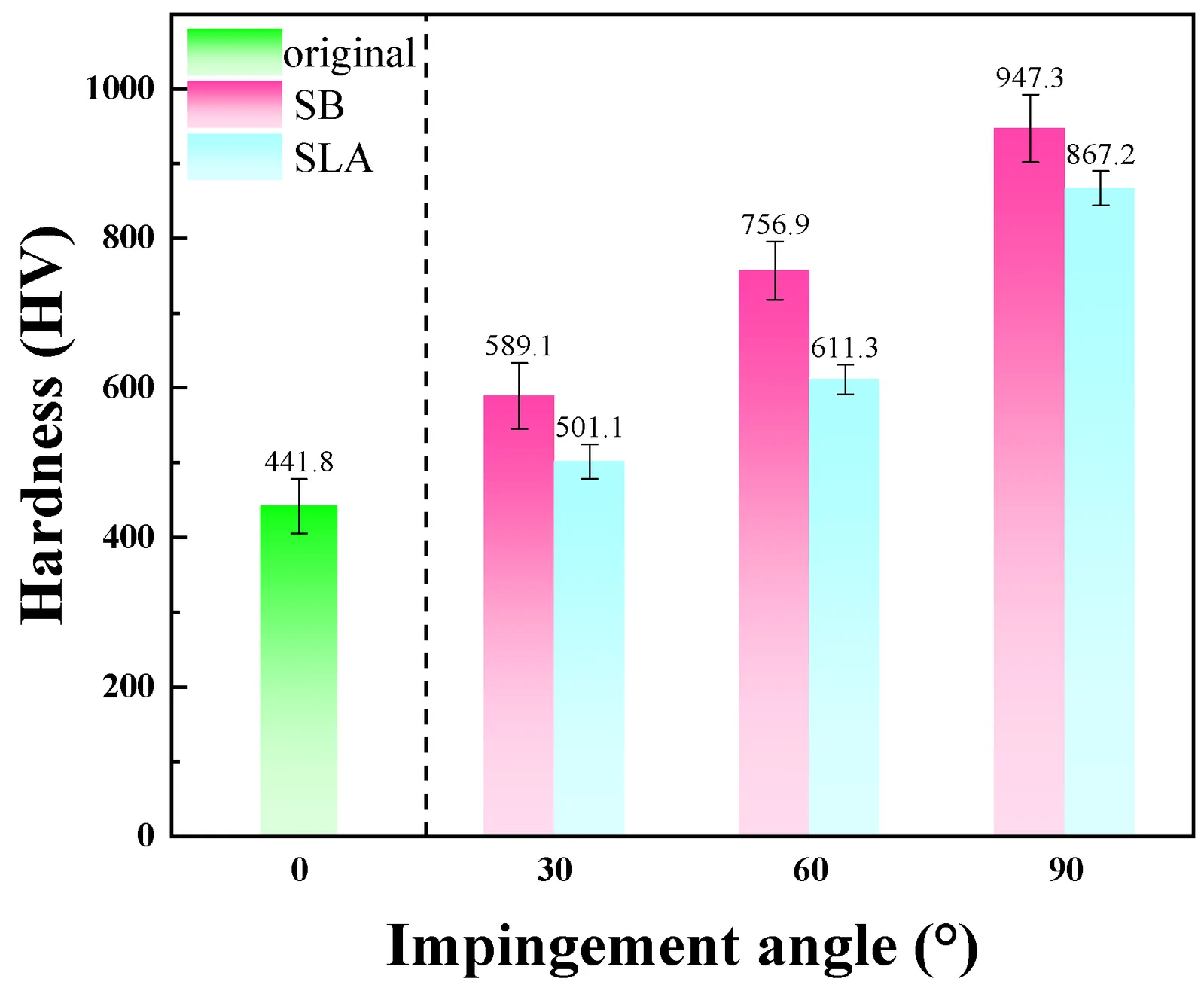
Surface microhardness of specimens under different treatment parameters.

**Figure 7:**
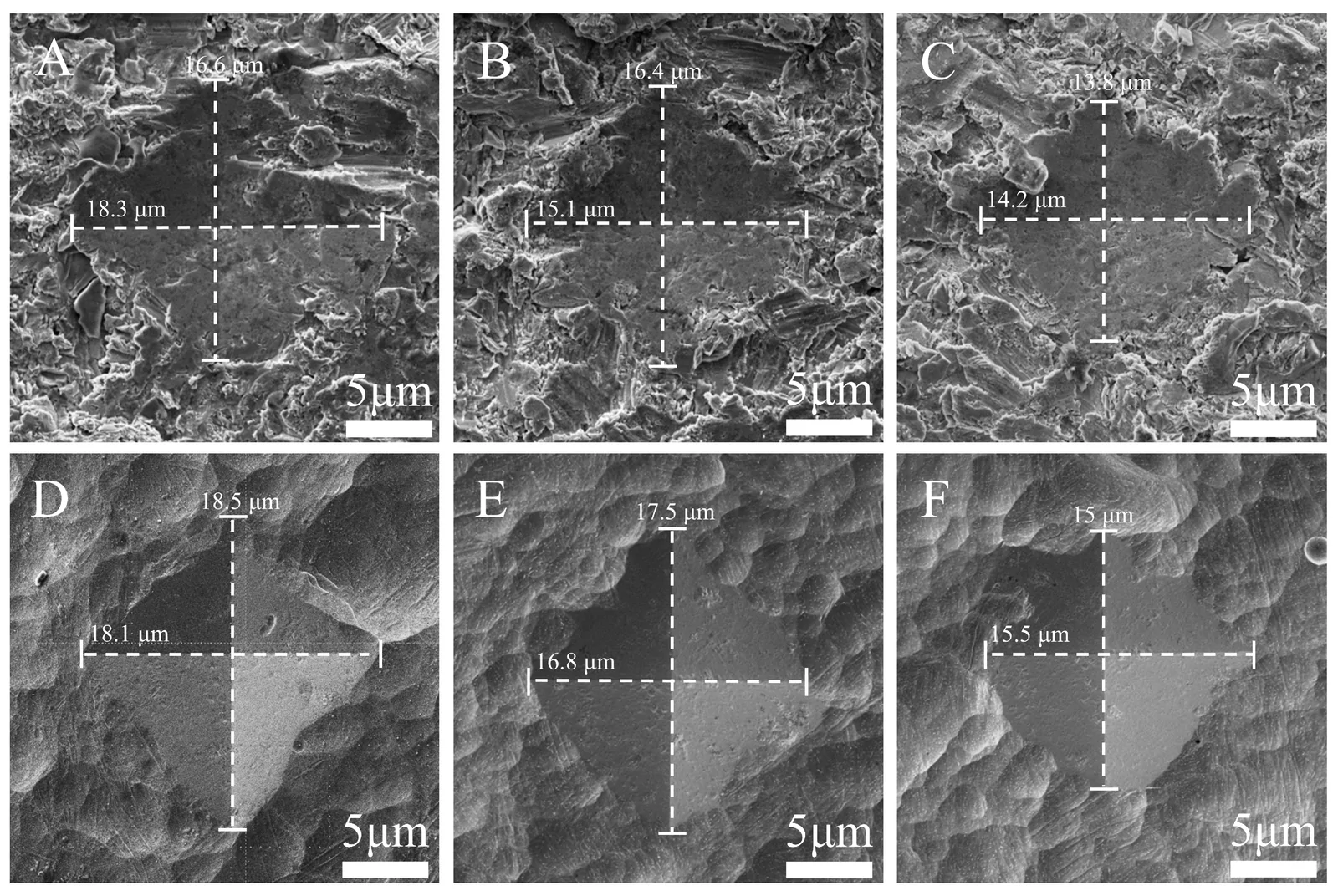
Surface morphologies of Vickers indentations on differently treated specimens (**A**) SB30; (**B**) SB60; (**C**) SB90; (**D**) SLA30; (**E**) SLA60; and (**F**) SLA90.

Mechanical energy input during blasting not only removes material from the outermost surface layer but also modifies the mechanical properties of the subsurface layer [34]. The microhardness results shown in Figure 6 and Figure 7 reflect the influence of energy distribution under different sandblasting angles on the surface hardness of Ti-6Al-4V. The experimental results show that single sandblasting remarkably increases the surface microhardness of all groups compared with the original state, with hardness values following the trend: 90° > 60° > 30°. This result is in good agreement with the mass loss results. For the sample blasted at 90°, the abrasive kinetic energy is mainly converted into normal impact force, and the intense vertical impact triggers substantial plastic deformation in the surface layer of Ti-6Al-4V [35]. During repeated impact, the surface material undergoes lattice distortion, dislocation multiplication, and dislocation entanglement, generating a pronounced work-hardening layer and the highest microhardness. In contrast, 30° sandblasting produces a larger tangential force. Most kinetic energy is consumed by surface cutting, ploughing, and material removal, so the normal force transmitted to the subsurface is relatively weak. The resultant work-hardened layer is thinner, corresponding to the least hardness increment. The 60° sandblasted sample shows a moderate hardening effect between the other two groups.

For samples subjected to the combined sandblasting and acid pickling treatment, the microhardness of each group decreases moderately compared with the corresponding as-sandblasted state, whereas the hardness ranking of 90° > 60° > 30° remains unchanged [36]. This observation suggests that acid pickling may partially remove or relax the mechanically affected outer surface generated during sandblasting, while the angle-dependent hardening effect is not fully eliminated. The outermost material layer undergoes the most severe impact, shear, and plastic deformation during sandblasting. Despite its high hardness, this layer typically contains inherent defects such as microcracks, material folding, plastic lips, and embedded abrasive particles. The mixed HF–HNO_3_ aqueous solution may preferentially attack exposed high-energy defective regions, including sharp protrusions, plastic lips, loose chips, and locally deformed surface features. This process can partially remove or relax the mechanically affected outer surface generated during sandblasting [37]. The retained hardness ranking suggests that the sandblasting-induced mechanical effect may extend beneath the immediate surface. Particularly for samples blasted at 90° and 60°, which experience stronger normal impact, a relatively high level of subsurface hardening is retained after acid pickling. In summary, the combined sandblasting–acid pickling treatment improves surface cleanliness and eliminates potential defects while preserving the surface strengthening effect introduced by sandblasting, achieving a favorable balance between surface purification and mechanical enhancement.

### 3.3. Roughness Analysis

**Figure 8:**
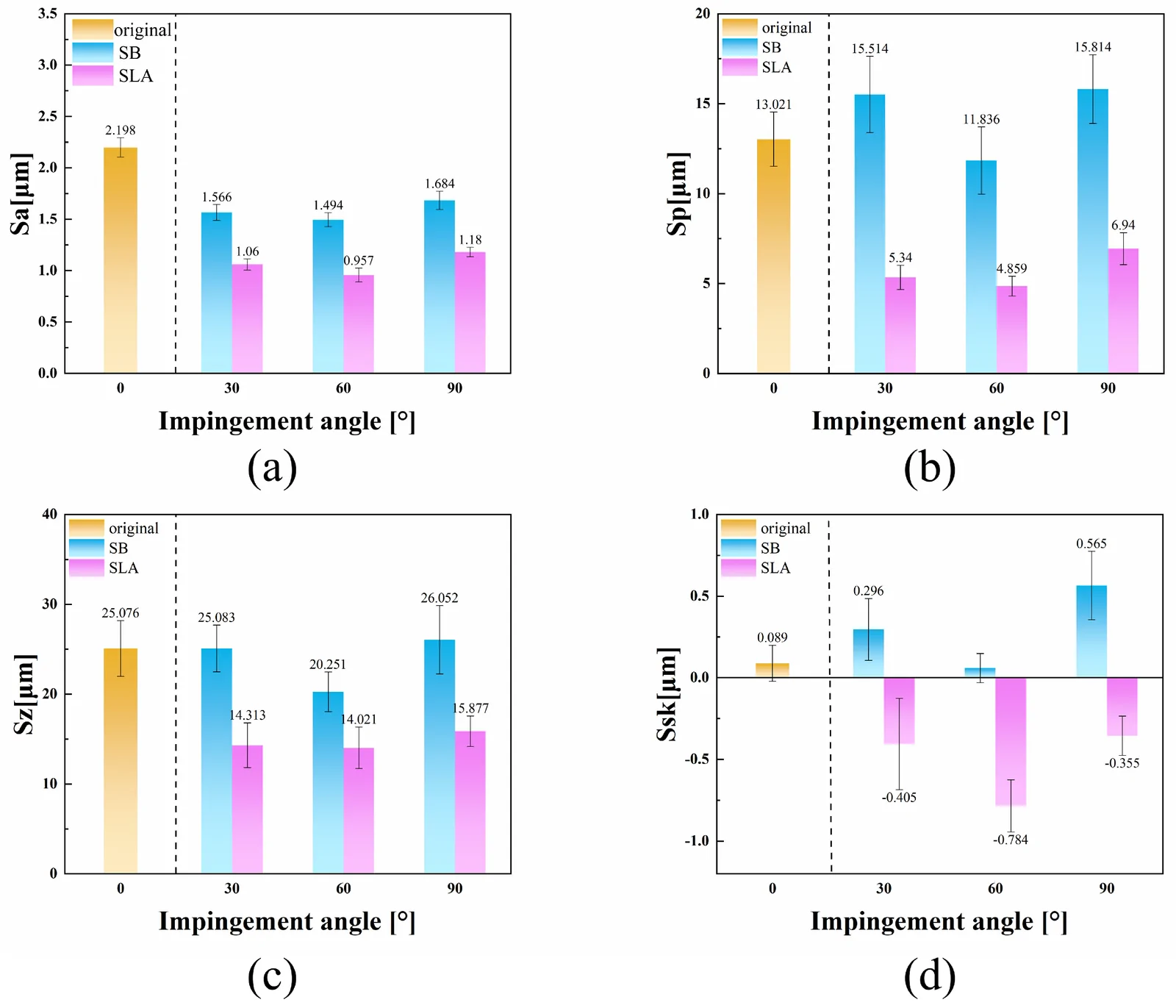
Surface roughness parameters of specimens under different treatment conditions: (**a**) Sa; (**b**) Sp; (**c**) Sz; and (**d**) Ssk.

**Figure 9:**
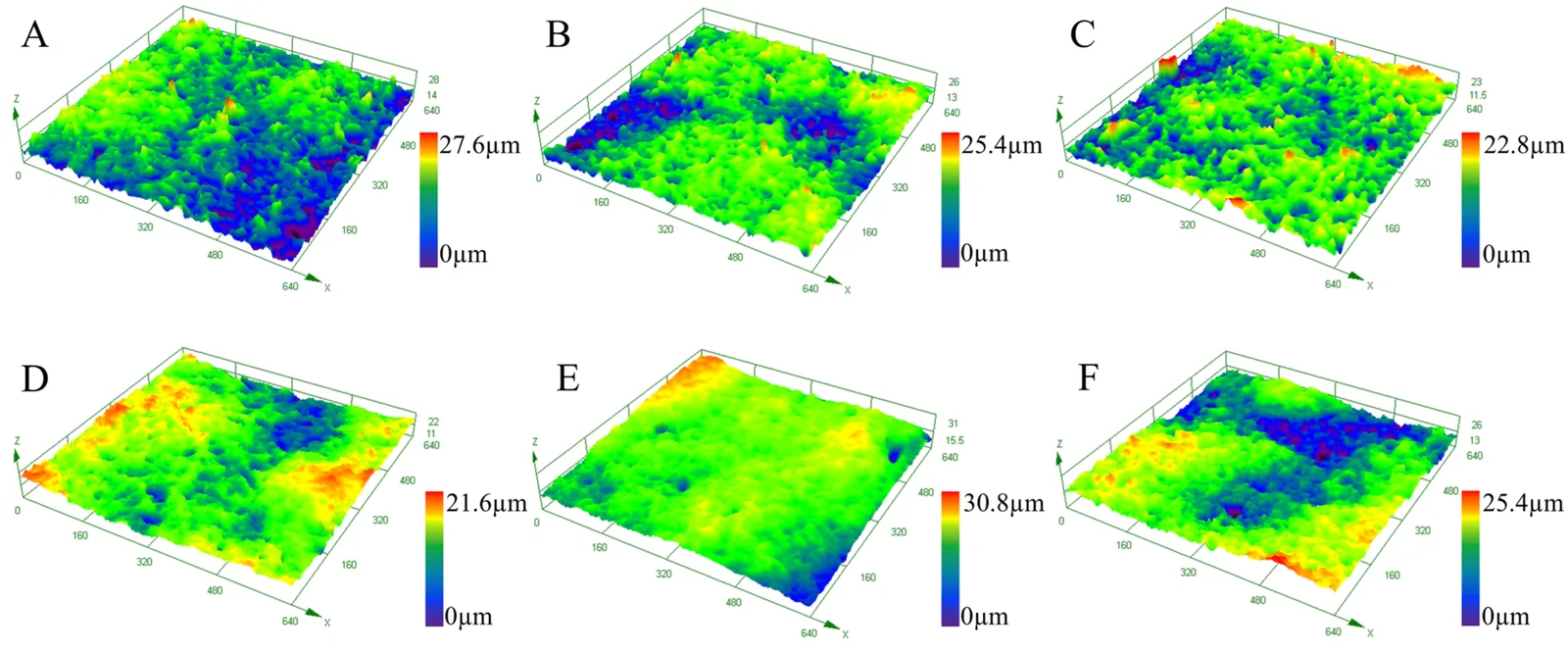
Surface three-dimensional morphology before and after sandblasting and pickling at different angles: (**A**): SB30; (**B**): SB60; (**C**): SB90; (**D**): SLA30; (**E**): SLA60; (**F**): SLA90.

Based on mass loss and microhardness test results, roughness testing and three-dimensional morphology observation were further combined to clarify the effects of sandblasting angles on the surface morphology of SLM Ti-6Al-4V, as shown in Figure 8 and Figure 9. The original sample presented the highest surface roughness, with a Sa value of 2.198 μm. This is mainly attributed to defects formed during SLM forming, such as adhered powders, spheroidized particles, uneven melt tracks, and local spatter particles [38]. The three-dimensional morphology also shows obvious peak-valley fluctuations and irregular protrusions on the original surface, indicating that the untreated SLM surface has large height variations. After sandblasting, the roughness of all groups decreased significantly, indicating that SiO_2_ abrasive particles can effectively remove loose particles and sharp protrusions, and mitigate surface undulations [39]. Among the tested blasting angles, the 90° sandblasting group had the highest roughness of 1.684 μm; the 60° group had the lowest roughness of 1.494 μm; and the 30° group had a roughness of 1.566 μm. When combined with the three-dimensional morphology results, the mass loss data indicate that 30° sandblasting leads to greater material removal. Nevertheless, the large tangential force of abrasive particles at a low angle tends to generate oriented grooves and local ploughing marks, preventing further reduction in roughness. For samples treated at 90°, normal impact played a dominant role. Although some particles on the original surface could be removed, numerous impact pits and local plastic deformation regions emerged, resulting in considerable surface height variations. In contrast, sandblasting at 60°combines cutting removal and normal impact, and can smooth surface peaks while avoiding deep impact pits from intense vertical impact. Therefore, the surface three-dimensional morphology is the most uniform and the roughness is the minimum. The combined data indicate that the lowest roughness at 60° stems from a trade-off between two competing mechanisms: a sufficient residual tangential component to remove adhered particles and surface peaks, coupled with a substantial yet not purely normal component that promotes local flattening without producing the dense array of deep impact pits observed at 90°. In contrast, the 30° condition preserves more pronounced directional grooving, while the 90° condition maximizes normal indentation and local height variation.

Subsequent acid pickling further reduced the roughness of samples from all blasting groups. The 60° sandblasting–acid-pickling group exhibited the lowest roughness of 0.957 μm, followed by the 30° sandblasting–acid-pickling group at 1.060 μm, while the 90° sandblasting–acid-pickling group remained the highest roughness of 1.180 μm. The three-dimensional morphology results also show that the surface peak-valley fluctuation is further reduced after acid pickling, indicating that the mixed HF-HNO3 aqueous solution preferentially dissolves residual surface protrusions, loose deformed layers and fine adhered particles after sandblasting, which further flattens the surface [40]. Notably, the roughness ranking across different angles remains unchanged after acid pickling, with the 60° group being the lowest and the 90° group the highest. This indicates that acid pickling can reduce the overall surface undulations, yet it cannot fully eliminate the initial morphology differences caused by sandblasting angle.

In addition to Sa, the parameters Sz, Sp, and Ssk were adopted to further characterize the surface height distribution. After sandblasting, the SB60 group exhibited the lowest Sz and Sp values, whereas SB30 and SB90 yielded comparatively higher extreme peak heights. This discrepancy is attributed to material accumulation along cutting grooves under the 30° condition and plastic ridges surrounding impact pits under the 90° condition. The results demonstrate that 60° sandblasting delivers a more balanced combination of asperity removal and impact-induced deformation.

Subsequent acid pickling significantly reduced Sz and Sp across all sandblasted specimens, confirming the effective attenuation of sharp protrusions, burrs, and plastic ridges. The SLA60 group maintained the lowest values, indicating the most favorable overall surface leveling performance. After sandblasting, Ssk values were positive or near zero, reflecting symmetrical or peak-dominated height distributions; following acid pickling, by contrast, they shifted to negative values. This transition indicates that acid pickling preferentially etches away prominent surface peaks, thereby increasing the relative contribution of residual pits and valleys. Overall, the sandblasting angle determines the initial peak–valley surface characteristics, while subsequent acid pickling further smooths the surface without completely eliminating the morphological features inherited from sandblasting [41].

### 3.4. Micro-Morphology, Elemental Composition, and Embedded-Particle Analysis

**Figure 10:**
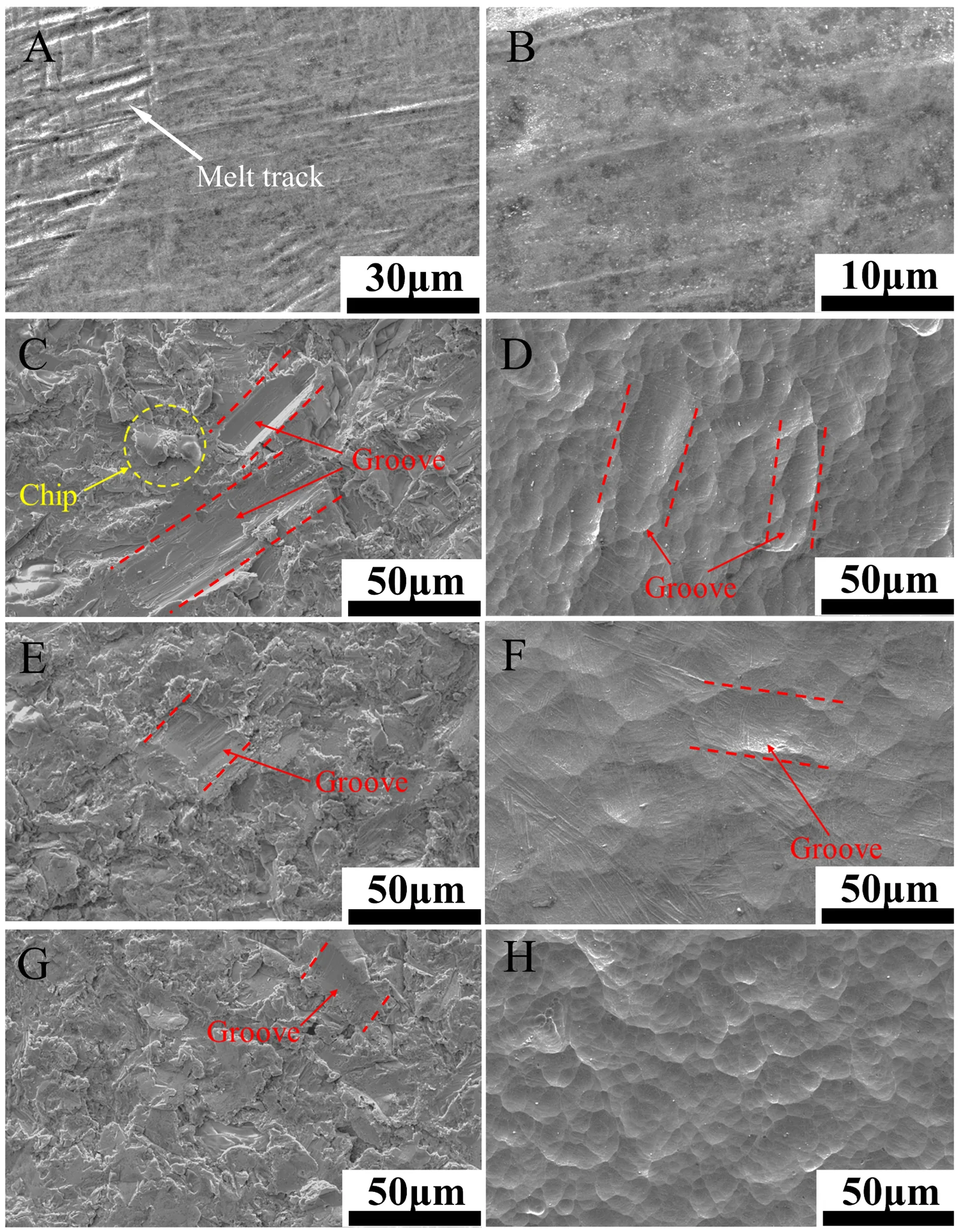
Surface micro-morphology before and after sandblasting and acid pickling at different angles: (**A**,**B**): as-built specimen at different magnifications; (**C**): SB30; (**D**): SLA30; (**E**): SB60; (**F**): SLA60; (**G**): SB90; and (**H**): SLA90. Representative melt tracks, chips, and groove features are indicated by arrows and dashed lines.

SEM observations revealed clear differences in surface microtopography between the as-built and treated specimens. As shown in Figure 10, the as-built surface exhibited overlapping, undulating melt tracks, reflecting the layer-by-layer melting and solidification characteristics of the SLM process. At higher magnification, numerous partially melted and adhered powder particles were observed on the surfaces and boundaries of the melt tracks. These features result in a highly heterogeneous surface with pronounced local protrusions, which accounts for the relatively high roughness of the as-built specimen.

After sandblasting, the local mechanical effects associated with different impact angles were distinctly reflected in the surface morphology. Abrasives deliver a dominant tangential force at 30°, so the surface undergoes predominant micro-cutting and ploughing. Corresponding SEM micrographs display distinct oriented cutting traces and continuous grooves, with residual metallic chips partially attached to groove edges. As the sandblasting angle increases to 90°, the action mode of the abrasive particles on the surface gradually changes from tangential shearing to normal impact. Accordingly, the surface morphology shifts from ploughing-dominated grooves to densely distributed impact pits. The surface of the 90° sandblasted sample shows numerous compact impact pits and local plastic deformation zones, accompanied by irregular plastic ridges around pit perimeters. This indicates that vertical impact causes obvious plastic flow and work hardening in the surface layer, which agrees well with the maximum microhardness observed in this group. In comparison, the 60° sandblasted sample exhibits a hybrid morphology containing both cutting traces and impact pits, demonstrating a balanced effect of tangential material removal and normal impact at this angle [42,43,44].

Considerable variations in microtopography are observed for all groups after subsequent acid pickling. Combined with the aforementioned hardness reduction in sandblasting–acid-pickling groups, it can be inferred that the HF-HNO_3_-H_2_O mixed acid solution selectively etches high-energy surface regions induced by sandblasting. Under relatively mild etching conditions, the mixed acid rarely triggers severe uniform corrosion on the Ti-6Al-4V substrate; instead, it preferentially dissolves sharp protrusions, plastic lips, loose cutting chips, and high-strain surface layers formed during sandblasting [45,46].

The SEM images confirmed that sharp burrs and loose particles were greatly diminished after acid pickling, and the edges of grooves and impact pits became rounded, leading to improved overall surface integrity. Meanwhile, the typical morphological features formed by sandblasting are well preserved. The SLA30 samples still possessed long, continuous ploughing grooves; the SLA90 samples retained well-defined discrete impact pits; and the SLA60 samples exhibited the most homogeneous surface morphology. This indicates that acid pickling cannot fully eliminate the surface differences caused by sandblasting. Instead, while removing loose surface defects and contaminants, this post-treatment further exposes, rounds, and reshapes the underlying morphology formed at different sandblasting angles [47]. This purification and reconstruction of micro-morphology not only explain why the original hardness ranking is maintained after acid pickling but also shapes the surface geometry that governs subsequent wettability performance.

**Figure 11:**
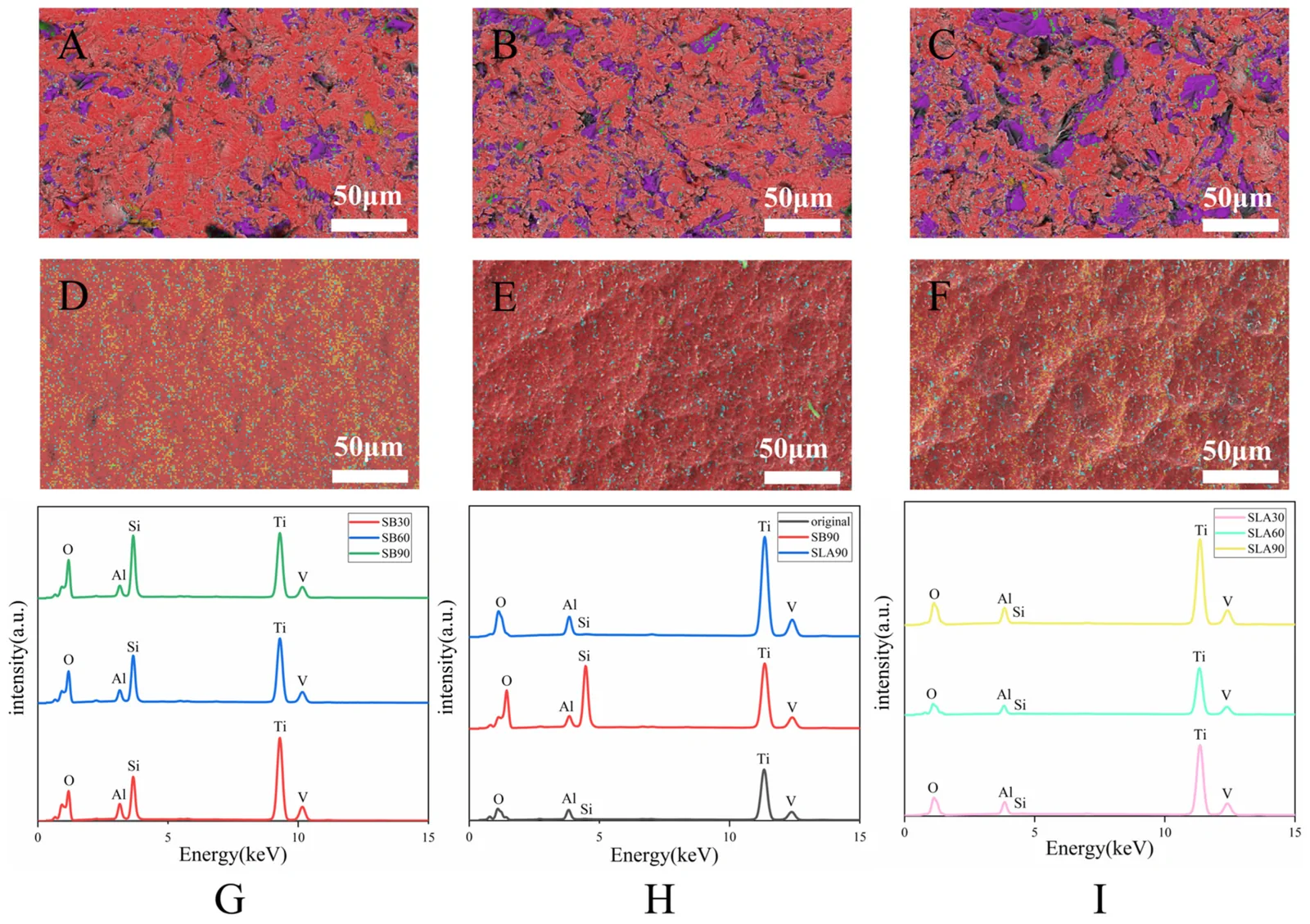
EDS elemental mapping and spectra of SLM-formed Ti-6Al-4V specimens under different treatment conditions: (**A**–**C**) SB30, SB60, and SB90; (**D**–**F**) SLA30, SLA60, and SLA90; (**G**) EDS spectrum of the sandblasted specimens; (**H**) EDS spectrum of the sandblasted and acid-pickled specimens; (**I**) comparison of the EDS spectra of the as-built, SB90, and SLA90 specimens. Red: Ti; purple: Si; cyan: V; green: O; brown: Al.

EDS analysis was performed to evaluate the elemental distribution and potential abrasive residues on the surfaces before and after acid pickling. As shown in Figure 11, distinct Si peaks were detected across all sandblasted specimens (SB30, SB60, and SB90), indicating that Si-containing particles remained on the Ti–6Al–4V surfaces after sandblasting. Given that quartz sand was used as the abrasive medium, these Si signals are mainly attributed to adhered or locally embedded SiO_2_ abrasive particles. Elemental mapping of SB90 further revealed that Si and O were concentrated in discrete surface regions rather than distributed uniformly, consistent with residual abrasive particles trapped within impact pits, grooves, and plastically deformed surface features. By comparison, Ti, Al, and V correspond primarily to the alloy matrix, although their apparent distributions are affected by the pronounced surface relief and the shielding effect of residual particles.

Following subsequent acid pickling, the Si signal on the SLA90 surface decreased markedly, approaching the EDS background level. The corresponding Si elemental map showed only sparse isolated signals, while the distributions of Ti, Al, and V became more continuous and homogeneous. These results confirm that the HF–HNO_3_ solution effectively removes most loosely adhered and near-surface SiO_2_ residues, along with burrs and plastically deformed protrusions generated during sandblasting. The O signal remained detectable after acid pickling; this is likely attributable to the rapid reformation of a titanium oxide passive layer upon exposure to water and air, rather than originating exclusively from residual quartz particles. Collectively, the EDS spectra and elemental mapping results demonstrate that acid pickling substantially improves the chemical cleanliness and elemental homogeneity of sandblasted surfaces.

**Figure 12:**
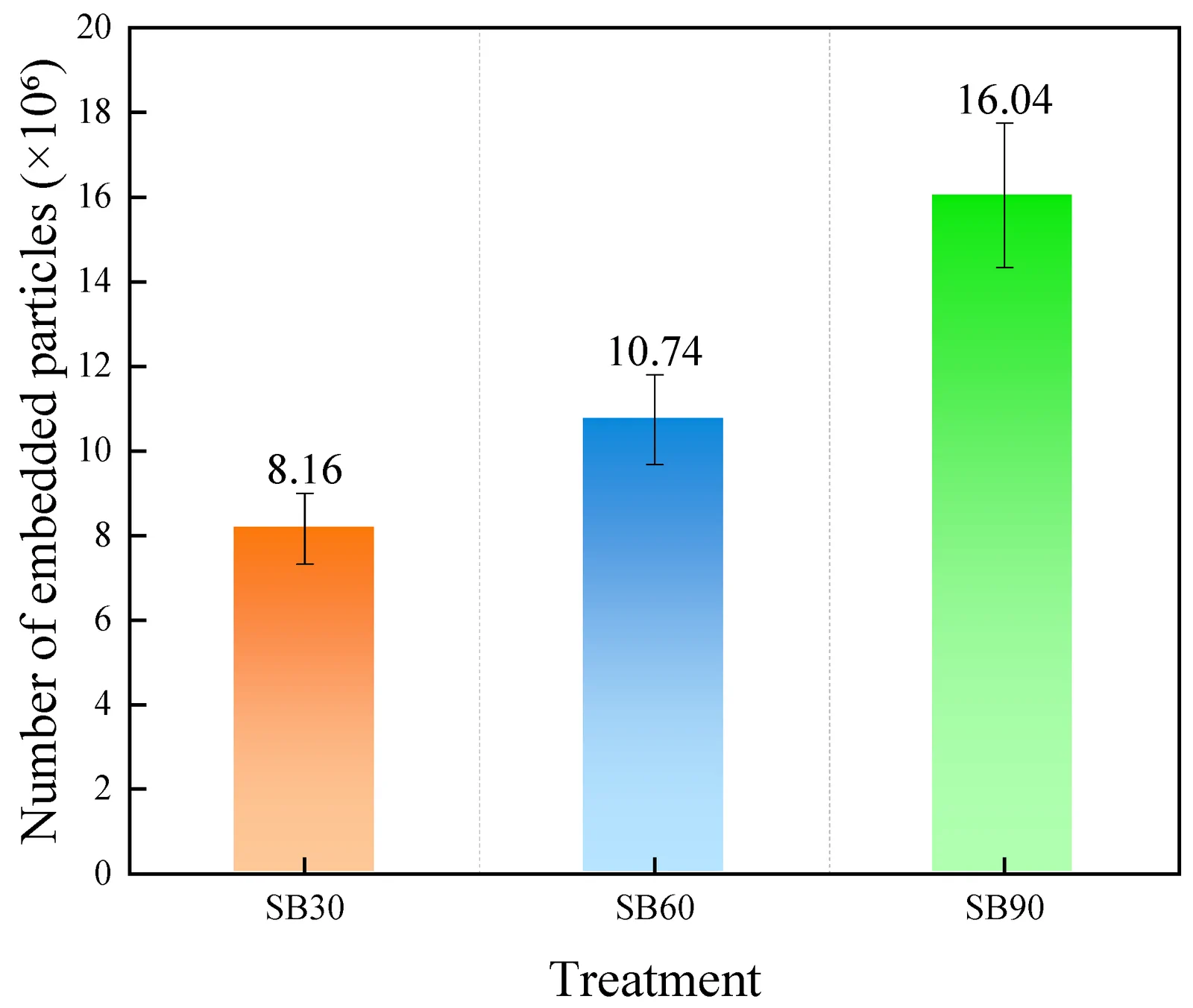
Number of embedded particles at different sandblasting angles.

As shown in Figure 12, the equivalent number of embedded SiO2 particles in the SB30, SB60, and SB90 specimens gradually increased with increasing sandblasting angle. Compared with SB30, the equivalent particle numbers of the SB60 and SB90 specimens increased by approximately 31.6% and 96.6%, respectively. This indicates that as the sandblasting angle increases, the normal component of the abrasive-particle velocity becomes stronger, making the particles more likely to be pressed into surface pits, plastic flaps, and local folded regions, thereby increasing the amount of surface embedment. This trend is consistent with the aforementioned microhardness results, in which the SB90 specimen exhibited both the highest number of embedded particles and the highest microhardness.

After acid pickling, under the same SEM/EDS observation conditions and particle identification criteria, no SiO_2_ particles satisfying the statistical threshold were detected on the surfaces of the SLA30, SLA60, and SLA90 specimens. This indicates that acid pickling can effectively remove visible embedded abrasive particles above the specified detection limit.

### 3.5. Surface Hydrophilicity Analysis

**Figure 13:**
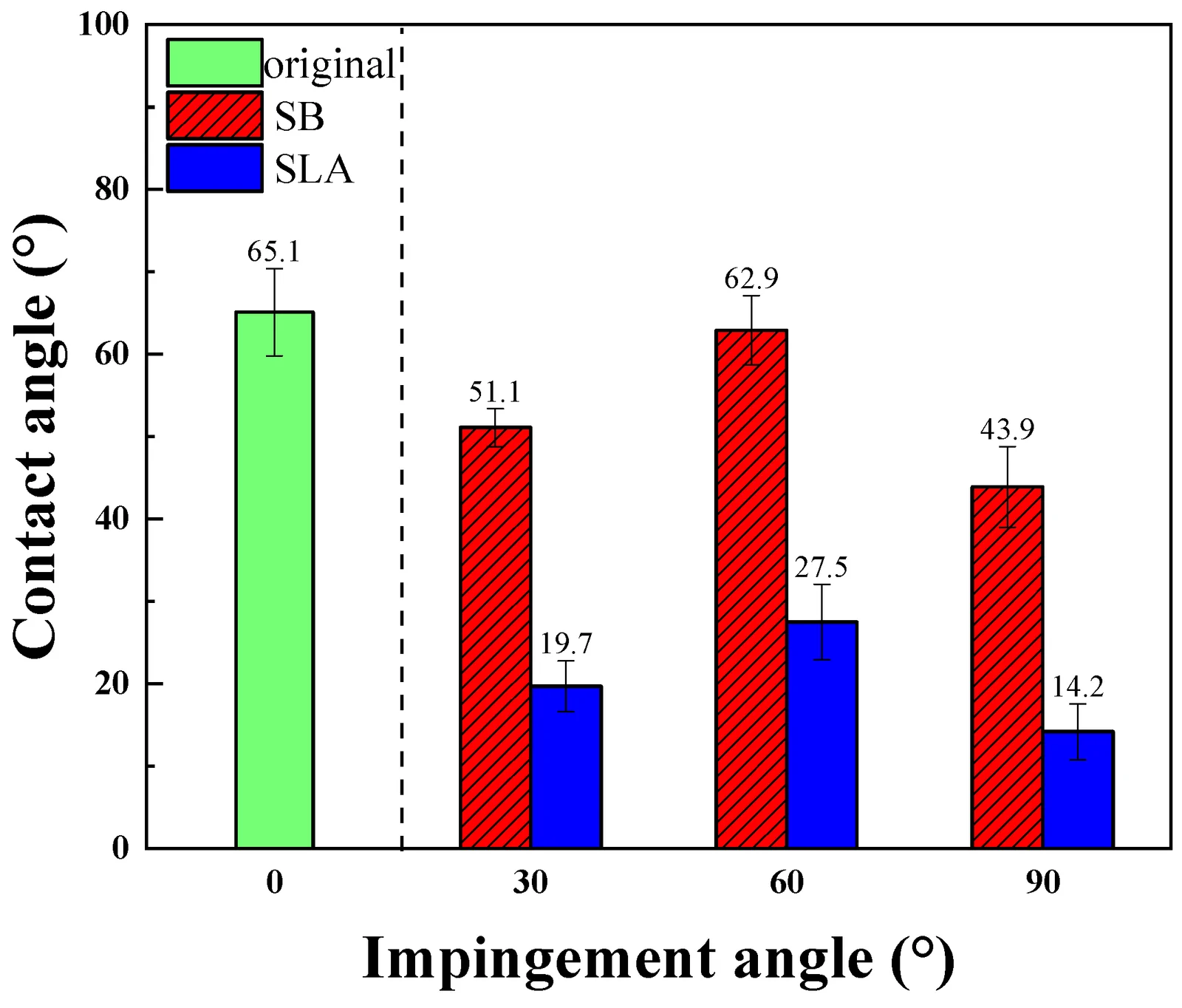
Surface contact angles of specimens under different treatment parameters.

**Figure 14:**
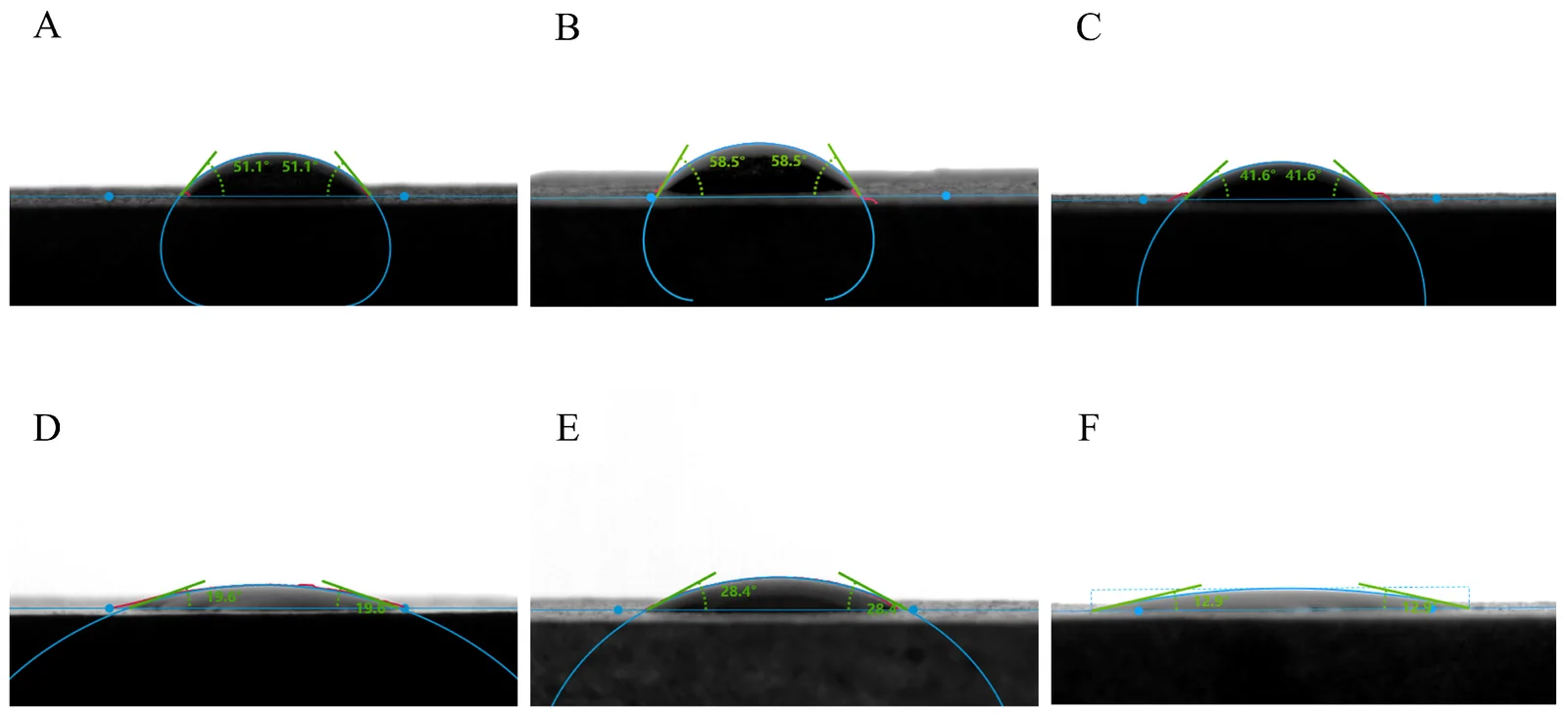
Contact angle images before and after sandblasting and acid pickling at different angles: (**A**): SB30; (**B**): SB60; (**C**): SB90; (**D**): SLA30; (**E**): SLA60; (**F**): SLA90.

The above changes in surface roughness and microtopography further influence the macroscopic wetting performance of the samples. Figure 13 and Figure 14 present the contact angle test results. Droplet spreading behavior under different surface modification states is highly dependent on surface microtexture and chemical composition. The as-built sample showed the largest contact angle, corresponding to poor wettability. This is primarily related to abundant irregular spheroidized particles, adhered powders, and macroscopic protrusions on the original SLM-formed surface. These disordered surface structures can easily trap air at the solid–liquid interface, keeping droplets in a Cassie–Baxter partially wet state and restricting their spreading [48]. After a single sandblasting treatment, the contact angles across all groups decreased significantly, indicating that sandblasting can remove part of the original surface defects and contaminant layers and improve surface wettability. Meanwhile, contact angle variations under different sandblasting angles are closely correlated with surface roughness and microtopographical features. The 90° sandblasted sample (90° SB) contains more microscopic impact pits and local undulations, which enlarge the actual solid–liquid contact area. In accordance with the Wenzel wetting model, when a material surface tends to be hydrophilic, increased surface roughness further enhances hydrophilicity [49]. Therefore, the 90° sandblasted sample showed the lowest contact angle among the sandblasted groups. In contrast, the 60° sandblasted sample (60° SB) has a relatively smooth and uniform surface morphology with the minimum roughness, leading to a weaker Wenzel amplification effect; thus, its contact angle is the highest among the sandblasted groups. The contact angle of the 30° sandblasted sample falls between the above two.

After the combined sandblasting and acid pickling treatment, all groups (SB + AP) showed a further reduction in contact angle compared with their sandblasted counterparts. The overall trend remains unchanged: the 90° group had the smallest contact angle and the 60° group the largest. This indicates that acid pickling enhances overall wettability but cannot fully eliminate the surface morphology differences caused by varying sandblasting angle. This phenomenon arises from the synergistic effect of chemical activation and geometric capillarity [50,51]. On the one hand, HF-HNO_3_-H_2_O mixed acid can remove residual contaminants, unstable oxide layers, and partially loose deformation layers after sandblasting, exposing fresh Ti-6Al-4V substrate and reforming a relatively clean and dense TiO_2_ passive film. The newly formed oxide layer may be enriched with polar hydroxyl groups (-OH), thereby increasing surface free energy and enhancing interaction with water molecules [52]. On the other hand, acid pickling dissolves and rounds groove edges, impact pit edges, and sharp protrusions after sandblasting, allowing the 30° group to form continuous micro-cutting grooves and the 90° group to retain distinct micropit structures. These microscale grooves and pits act as capillary channels and accelerate droplet infiltration and spreading. Therefore, the further decrease in contact angles after the sandblasting–acid-pickling composite treatment results from the combined effects of surface chemical activation, roughness regulation, and capillary spreading [53,54]. Such modified surfaces, which possess relatively high surface free energy and specific microscale capillary structures, offer favorable conditions for the surface functionalization of SLM Ti-6Al-4V in interfacial wetting regulation and relevant engineering applications.

### 3.6. Residual Stress Analysis

**Figure 15:**
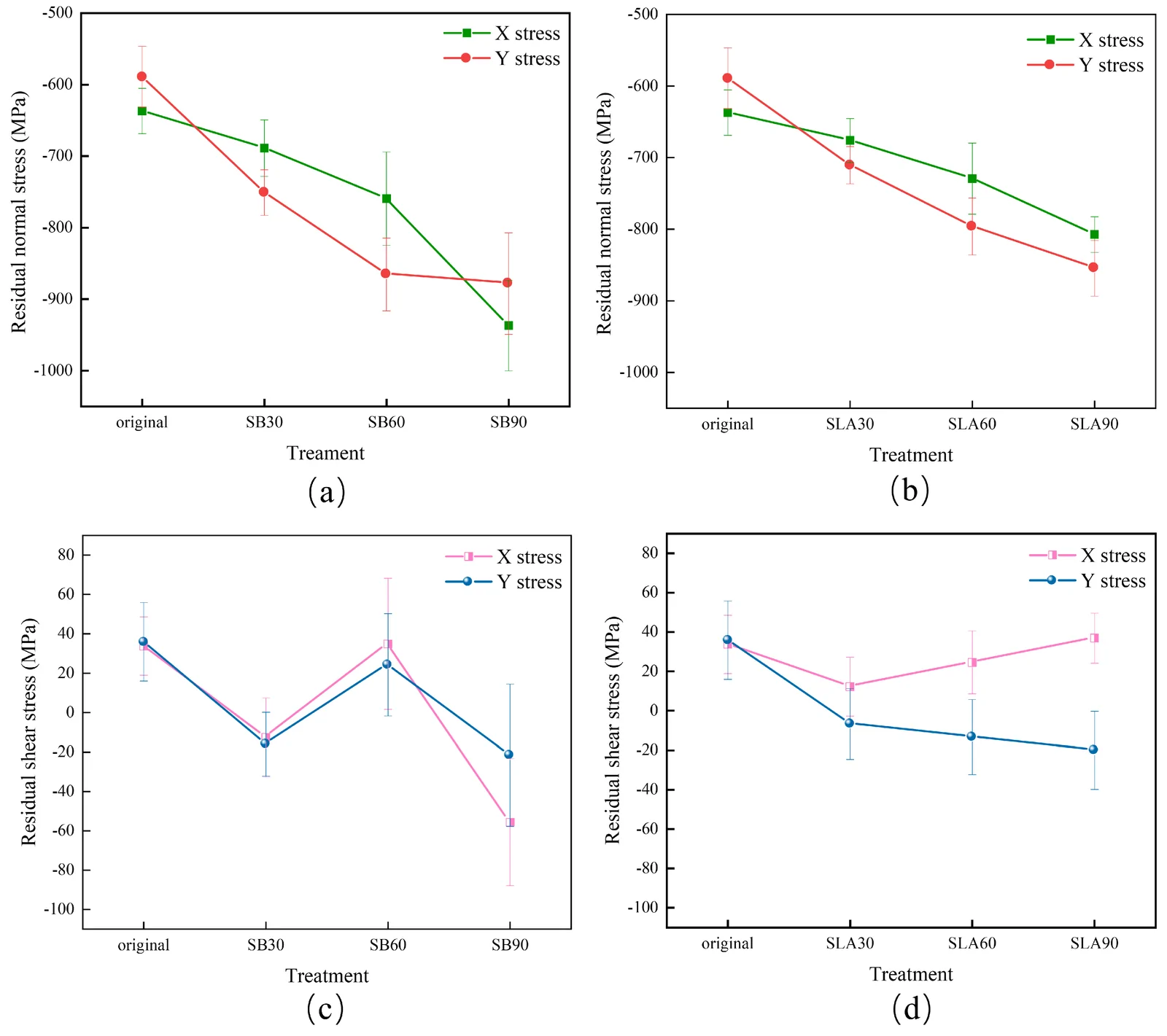
Residual stress variation of SLM Ti-6Al-4V specimens under different surface treatment conditions: (**a**) residual normal stresses in the X and Y directions for specimens sandblasted at different angles; (**b**) residual normal stresses in the X and Y directions after acid pickling; (**c**) residual shear stresses in the X and Y directions for specimens sandblasted at different angles; (**d**) residual shear stresses in the X and Y directions after acid pickling.

As shown in Figure 15, both the as-built and surface-treated specimens exhibited negative residual normal stresses in the X and Y directions, indicating that the specimen surfaces were generally in a state of residual compressive stress. The residual normal stresses of the as-built specimen were −637.16 ± 31.74 MPa and −589.20 ± 42.66 MPa in the X and Y directions, respectively. After sandblasting, the magnitudes of residual compressive stress in both directions increased significantly and showed a general upward trend with rising sandblasting angle. The residual normal stresses of the SB30, SB60, and SB90 specimens in the X direction were −689.00 ± 39.40, −759.67 ± 65.33, and −937.03 ± 63.11 MPa, respectively, while those in the Y direction were −750.96 ± 31.75, −865.38 ± 50.92, and −878.32 ± 70.89 MPa, respectively. Among them, the SB90 specimen exhibited the highest level of residual compressive stress, with the magnitudes of residual normal stress in the X and Y directions increasing by approximately 47.1% and 49.1%, respectively, compared with the as-built specimen. The quantity of embedded abrasive particles and the magnitude of residual compressive stress showed similar increasing trends with increasing sandblasting angle, indicating that both were closely related to the progressively enhanced normal impact effect. Higher normal impact energy, on the one hand, promoted more intense indentation deformation and plastic flow in the near-surface layer, thereby forming a higher level of residual compressive stress; on the other hand, it also increased the probability that abrasive particles become embedded within surface pits, grooves, and plastically deformed regions.

The above changes are mainly attributed to variations in the normal and tangential components of the abrasive-particle impact load at different sandblasting angles. At smaller sandblasting angles, the abrasive particles primarily produce tangential ploughing and micro-cutting effects, and the surface-layer deformation is dominated by directional material removal and relatively shallow plastic deformation. As the sandblasting angle increases, the normal component of particle velocity and the normal impact energy gradually increase, resulting in more severe indentation deformation, plastic flow, and dislocation accumulation in the near-surface region. After impact unloading, the plastically deformed surface layer is constrained by the underlying elastic matrix, thereby generating a higher level of residual compressive stress [55]. Therefore, the residual compressive stresses in both measurement directions generally followed the decreasing order of SB90, SB60, SB30, and the as-built condition. This trend is consistent with the microhardness results, in which the 90° sandblasted specimen exhibited the highest hardness, further indicating that a larger sandblasting angle can promote more pronounced near-surface plastic deformation and work hardening [56].

In addition to residual normal stresses, all specimens also exhibited a certain degree of residual shear stress. The residual shear stresses obtained from the as-built specimen in the X and Y measurement directions were 33.8 ± 14.8 MPa and 35.9 ± 19.9 MPa, respectively. After sandblasting, the residual shear stress did not show the same monotonic dependence on sandblasting angle as the residual normal stress. The residual shear stresses of the SB30 specimen in the X and Y directions were −12.6 ± 20.1 MPa and −16.0 ± 16.2 MPa, respectively; those of the SB60 specimen were 34.9 ± 33.3 MPa and 24.3 ± 26.0 MPa, respectively; and those of the SB90 specimen were −55.6 ± 32.2 MPa and −21.6 ± 36.1 MPa, respectively. Compared with the residual normal stresses, the magnitudes of the residual shear stresses were much smaller, and some measured values were of the same order of magnitude as their fitting uncertainties, indicating that their variations were more susceptible to local surface morphology, sandblasting path, random abrasive incidence, and measurement direction.

The different variation trends of residual normal stress and residual shear stress are mainly due to their different formation mechanisms. Residual normal stress is primarily governed by the normal impact of abrasive particles, indentation-induced plastic deformation of the surface layer, and matrix constraint; therefore, it generally increases with increasing sandblasting angle. In contrast, residual shear stress is more sensitive to the tangential velocity component of abrasive particles, the sandblasting motion direction, and surface anisotropy. In this study, sandblasting was performed along the X direction. In particular, under oblique incidence at 30° and 60°, the abrasive particles had a relatively obvious tangential motion component in the X direction, which could readily induce directional ploughing, material dragging, and local plastic flow. However, stronger tangential action may also intensify surface material removal, causing some high-shear deformation regions to be directly removed. Therefore, the residual shear stress retained in the surface layer does not necessarily increase monotonically with the tangential velocity component. For 90° sandblasting, although the tangential velocity component is relatively small under ideal normal incidence, particle rebound, local surface inclination, movement of the spray gun along the X direction, and asymmetric plastic flow around pits may still generate a certain level of residual shear stress. Thus, fluctuations in residual shear stress are more appropriately interpreted as the combined results of sandblasting directionality, local impact non-uniformity, and surface morphological anisotropy.

After subsequent acid pickling, the residual normal stresses of the SLA90 specimen in the X and Y directions were −807.42 ± 24.92 MPa and −854.39 ± 38.84 MPa, respectively. Compared with those of SB90, the magnitudes of residual compressive stress in the X and Y directions decreased by approximately 13.8% and 2.7%, respectively. This decrease may be attributed to the preferential dissolution of the highly deformed near-surface material where residual compressive stress was concentrated, together with a certain degree of local stress relaxation. Nevertheless, the SLA90 specimen still retained residual compressive stresses in both directions that were markedly higher than those of the as-built specimen, indicating that acid pickling only removed or relaxed part of the highly stressed outer surface layer and did not completely eliminate the near-surface mechanical effects induced by sandblasting [57]. The residual shear stresses of the SLA90 specimen in the X and Y directions were 36.9 ± 12.7 MPa and −20.0 ± 19.8 MPa, respectively, indicating that a certain directional shear-stress state remained on the surface after acid pickling. The change in shear-stress sign relative to SB90 may be associated with the selective dissolution of local highly deformed regions, groove edges, and plastic flaps during acid pickling, a process that alters the local distribution and stress release pathways of the non-uniform near-surface stress field.

Overall, sandblasting can effectively increase the residual compressive stress on the surface of SLM-formed Ti-6Al-4V, with 90° sandblasting producing the most pronounced strengthening effect. Subsequent acid pickling induces partial relaxation of the residual normal stress, but does not completely eliminate the residual compressive-stress layer formed by sandblasting. Unlike residual normal stress, which exhibits an overall increasing trend, residual shear stress mainly presents directional fluctuations, reflecting the combined influence of the tangential action of abrasive particles, the sandblasting path, and local morphological non-uniformity. In combination with the microhardness results, the results collectively demonstrate that as the sandblasting angle increases, the enhanced normal impact and near-surface plastic deformation promote the formation of surface work hardening and residual compressive stress.

## 4. Discussion

Previous studies have established that post-processing is essential for improving the surface quality and service performance of SLM-fabricated Ti–6Al–4V, as the as-built surface typically features adhered powder particles, balling particles, and undulating melt tracks, which give rise to high surface roughness. A range of surface treatments, including sandblasting, acid etching, chemical polishing, shot peening, and laser shock peening, have been employed to mitigate surface defects and reduce surface roughness, enhancing wettability and mechanical properties. However, existing investigations have predominantly focused on blasting pressure, abrasive properties, acid formulation, and pickling duration. The influence of the sandblasting impact angle—particularly its role in governing the initial surface morphology, near-surface deformation, and the subsequent response to acid pickling—remains incompletely understood.

This study presents a hybrid surface modification strategy combining variable-angle sandblasting and acid pickling for selective laser melted (SLM) Ti–6Al–4V titanium alloy and systematically assesses the resulting surface performance. The main conclusions are summarized as follows:

(1) The sandblasting angle governs the balance between normal impact and tangential shear, thereby regulating material removal, surface work hardening and abrasive particle embedment. At 30°, cutting and ploughing are the dominant mechanisms, yielding the highest mass loss and the lowest amount of embedded particles, but the weakest hardening effect. At 90°, normal impact induces more extensive plastic deformation, resulting in the lowest mass loss, markedly increased particle embedment, and highest microhardness, while the 60° condition exhibits intermediate behavior.

(2) Sandblasting markedly reduces the surface roughness of SLM-fabricated Ti–6Al–4V by eliminating adhered powder particles, spheroidized particles, and sharp surface protrusions. Among the angles investigated, 60° achieves the optimal balance between material removal and impact-induced flattening, delivering the lowest surface roughness.

(3) Subsequent pickling in an HF–HNO_3_–H_2_O solution further smooths sandblasted surfaces by removing sharp protrusions and loose surface debris. All acid-pickled specimens exhibit reduced roughness and microhardness, with the 60° group achieving the most uniform and level surface. These findings are consistent with the partial removal or relaxation of the mechanically affected outer surface layer.

(4) The combined sandblasting–acid pickling treatment significantly enhances surface wettability. Sandblasting eliminates surface defects and modifies the surface microtopography, while acid pickling cleans and activates the surface. The remaining microgrooves and micropits further facilitate liquid spreading via capillary action.

(5) All specimens exhibited compressive residual stress. The mean residual stress increased from approximately −613 MPa in the as-built state to −720, −813, and −908 MPa following sandblasting at 30°, 60°, and 90°, respectively. After acid pickling, the mean value for the 90° group decreased to approximately −831 MPa but remained higher than that of the as-built specimen, indicative of partial stress relaxation without full elimination of the near-surface mechanical effects induced by sandblasting.

## 5. Conclusions

In summary, this study establishes a process–mechanism–property framework for the combined sandblasting and acid-pickling treatment of SLM Ti–6Al–4V. The key scientific insight is that the sandblasting angle controls the balance between tangential cutting and normal impact, thereby governing the initial surface morphology, near-surface mechanical state, and subsequent response to chemical etching. Acid pickling does not simply smooth the surface; rather, it modifies the mechanically generated topography by preferentially attenuating exposed peaks and removing surface residues while possibly retaining part of the near-surface mechanical strengthening effect.

The results further demonstrate that surface flatness, hardness, residual stress, and wettability cannot be optimized simultaneously by a single treatment condition. Therefore, the selection of processing parameters should be application-oriented and based on the required balance among geometric quality, mechanical strengthening, and interfacial performance. This work provides a mechanistic basis for tailoring the surface properties of additively manufactured Ti–6Al–4V through coordinated mechanical and chemical modification.

Future work will focus on validating the subsurface deformation and etching mechanisms through cross-sectional characterization and depth-resolved mechanical testing, together with long-term evaluations of wettability stability, corrosion resistance, fatigue behavior, and wear performance.

## Figures and Tables

**Table 1:** Chemical composition of Ti-6Al-4V (wt%).

Ti	Al	V	Fe	O	C	N	H
bal	6.28	3.95	0.032	0.076	0.013	0.012	0.0005

**Table 2:** Sandblasting and acid pickling process.

Impingement Angle	Acid Pickling	Lab Number
0	No	original1
0	No	original2
0	No	original3
30°	No	SB301
30°	No	SB302
30°	No	SB303
30°	Yes	SLA301
30°	Yes	SLA302
30°	Yes	SLA303
60°	No	SB601
60°	No	SB602
60°	No	SB603
60°	Yes	SLA601
60°	Yes	SLA602
60°	Yes	SLA603
90°	No	SB901
90°	No	SB902
90°	No	SB903
90°	Yes	SLA901
90°	Yes	SLA902
90°	Yes	SLA903

## Data Availability

The original contributions presented in this study are included in the article. Further inquiries can be directed to the corresponding author.
